# Atractylenolide-III suppresses lipopolysaccharide-induced inflammation via downregulation of toll-like receptor 4 in mouse microglia

**DOI:** 10.1016/j.heliyon.2021.e08269

**Published:** 2021-10-27

**Authors:** Ela Novianti, Goro Katsuura, Namiko Kawamura, Akihiro Asakawa, Akio Inui

**Affiliations:** a Department of Psychosomatic Internal Medicine, Kagoshima University Graduate School of Medical and Dental Sciences, Kagoshima, 890-8544, Japan; b Research Center for Biotechnology, Indonesian Institute of Sciences, Jl. Raya Bogor Km 46, Jawa Barat, 16911, Indonesia; c Drug Discovery of Next-Generation GcMAF, Kagoshima University Graduate School of Medical and Dental Sciences, Kagoshima, 890-8544, Japan; d Pharmacological Department of Herbal Medicine, Kagoshima University Graduate School of Medical and Dental Sciences, Kagoshima, 890-8544, Japan

**Keywords:** Atractylenolide-III, Mouse microglia, Cytokine, TLR4, p38 MAPK, JNK

## Abstract

Atractylenolide-III (AIII), a sesquiterpene compound isolated from the rhizome of *Atractylodes macrocephala,* has been reported to have anti-inflammatory effects in the peripheral organs. However, its effects on brain inflammation remain elusive. The present study investigated the effects of AIII on the response to lipopolysaccharide (LPS) in mouse microglia and clarified the underlying mechanism. In this study, treatment of MG6 cells with AIII (100 μM) significantly decreased the mRNA expression and protein levels of toll-like receptor 4 (TLR4). In addition, pretreatment of MG6 cells and primary cultured microglia cells with AIII (100 μM) significantly decreased the mRNA expression and protein levels of tumor necrosis factor-α, interleukin-1β, interleukin-6, inducible nitric oxide synthase, and cyclooxygenase-2 induced by LPS (5 ng/mL) without cytotoxicity. Subsequently, pretreatment with AIII significantly suppressed the phosphorylation of p38 mitogen-activated protein kinase (p38 MAPK) and c-Jun NH_2_-terminal kinase (JNK) after LPS stimulation in MG6 cells. These results showed that AIII downregulated TLR4 expression, leading to suppression of the p38 MAPK and JNK pathways, which in turn inhibited the production of pro-inflammatory cytokines and enzymes in LPS-stimulated microglia. Our findings, therefore, suggest the potential for AIII as a therapeutic agent for the treatment of brain inflammation, particularly in microglia-associated inflammation.

## 1 Introduction

Byakujutsu (Baizhu in Chinese), the rhizome of *Atractylodes macrocephala*, is used as an herbal medicine because of its broad biological functions, ranging from alleviating fatigue to anti-cancer effects. One of the main active compounds in Byakujutsu is atractylenolide-III (AIII), chemically a sesquiterpene lactone [1]. Previous reports revealed anti-inflammatory effects of AIII in the peripheral organs. For instance, in vitro studies demonstrated inhibitory effects of AIII on the production of tumor necrosis factor-α (TNF-α), interleukin-6 (IL-6), inducible nitric oxide synthase (iNOS), and cyclooxygenase-2 (COX-2) in lipopolysaccharide (LPS)-stimulated mouse macrophages [2, 3] and in 12-myristate 13-acetate- and calcium ionophore A2318-stimulated human mast cells [4]. Furthermore, oral administration of AIII in LPS-injected mice suppressed the serum levels of TNF-α and IL-6 [5]. However, the effects of AIII on brain inflammation and its underlying mechanisms remain to be elucidated. Intriguingly, a pharmacokinetic assessment results demonstrated that AIII exerts high permeability to the blood-brain barrier [6, 7], suggesting that this compound might regulate brain inflammation.

The central nervous system (CNS) consists of neurons and glial cells, including microglia, astrocytes, and oligodendrocytes as their major components. Both microglia and astrocytes play a pivotal role in brain inflammation because of expressing toll-like receptor 4 (TLR4) in these cells [8]. Accumulating evidence demonstrates that brain inflammation, caused by microglial overactivation, leads to cognitive impairment, anorexia, neuropsychiatric disorders, and neurodegeneration such as Alzheimer's disease (AD), Parkinson's disease (PD), and multiple sclerosis [9]. Both in vitro and in vivo studies have shown that LPS activates microglia in the brain through the stimulation of TLR4. Upon LPS stimulation, the downstream signaling cascade is activated and promotes the recruitment of the adapter protein myeloid differentiation primary response gene 88. This condition leads to the activation of nuclear factor kappa B (NF-κB) and mitogen-activated protein kinases (MAPKs) consisting of p38 MAPK, c-Jun NH_2_-terminal kinase (JNK), and extracellular signal-regulated kinase 1/2 (ERK1/2), which results in excess production of pro-inflammatory cytokines, including TNF-α, interleukin-1β (IL-1β) and IL-6, as well as iNOS and COX-2 [10,11].

In this study, we examined the effects of AIII on the response of mouse microglia to LPS and elucidated the mechanism. Our findings revealed that AIII suppressed the LPS-induced production of inflammatory cytokines and enzymes in mouse microglia through the downregulation of TLR4.

## 2 Materials and methods

### 2.1 Cell culture and treatments

MG6 cells [12, 13], an immortalized mouse microglia cell line (RCB 2403, RIKEN Cell Bank, Tsukuba, Japan), were cultured in Dulbecco's Modified Eagle Medium (DMEM; Thermo Fisher Scientific Inc., Waltham, MA, USA) containing 10% fetal bovine serum (FBS; Thermo Fisher Scientific Inc.) with 10 μg/mL insulin (Thermo Fisher Scientific Inc.), 100 μM β-mercaptoethanol (Nacalai Tesque, Kyoto, Japan), and 1% antibiotic-antimycotic solution (Thermo Fisher Scientific Inc.). Cells were seeded on 6-well plates (1 × 10^5^ cells/mL/well) and maintained in 95% humidified air and 5% CO_2_ at 37 °C for 3–4 days.

The mouse macrophage cell line RAW264.7 (ECACC 91062702, European Tissue Culture Collection, London, UK) was cultured in DMEM supplemented with 10% FBS and 1% antibiotic-antimycotic solution, seeded on 6-well plates (5 × 10^5^ cells/mL/well), and maintained in 95% humidified air and 5% CO_2_ at 37 °C for 3–4 days.

Primary cultured microglia cells (PMC) were obtained from 1-3 day-old C57BL/6J mouse brain according to previously reported methods [14]. Cells were seeded on 6-well plates (5 × 10^5^ cells/mL/well) for 3–4 days and maintained in 95% humidified air and 5% CO_2_ at 37 °C. All experiments in this study were approved by the Experimental Animal Research Committee of Kagoshima University (Approval number: MD19096).

The effects of AIII (Wako Pure Chemical Industries, Ltd., Osaka, Japan) on LPS (Merck, KGaA, Darmstadt, Germany) -induced inflammation were investigated as follows. To examine the effects of AIII on the expression of TLR4, MG6 cells were incubated with or without AIII (100 μM) for 1, 3, or 6 h. Additionally, MG6 cells were incubated with AIII (1, 10, and 100 μM) for 3 h to investigate the concentration dependence of the effects of AIII on TLR4 mRNA expression. Furthermore, RAW264.7 cells, MG6 cells, and PMC were pre-incubated with or without AIII (100 μM) for 3 h, followed by the addition of LPS (5 ng/mL) for 3 h. The cells and culture media were collected for analysis of cytokine and enzyme production. Phosphorylation of p38 MAPK, JNK, and NF-κB were examined by incubating MG6 cells with or without AIII (100 μM) for 3 h and then adding LPS (5 ng/mL) for 15, 30, and 60 min.

### 2.2 Reverse transcription-polymerase chain reaction (RT-PCR)

The mRNA expression of TLR4, TNF-α, IL-1β, IL-6, iNOS, and COX-2 was measured by RT-PCR as described previously [15]. Following the treatment, the cells were lysed and total RNA was isolated using RNeasy Mini Kit (QIAGEN, Hilden, Germany) and cDNA was synthesized using Verso cDNA Synthesis Kit (Thermo Fisher Scientific Inc.). Aliquots of diluted cDNA were amplified with FastStart SYBR Green Master (Roche Applied Science, Mannheim, Germany) in a final volume of 25 μl on Thermal Cycler Dice Real Time System (TAKARA BIO INC., Shiga, Japan). The RT-PCR schedule was 95 °C for 10 min followed by 40 cycles of 5 s at 95 °C, 30 s at 60 °C and 1 cycle of 15 s at 95 °C, 30 s at 60 °C and 15 s at 95 °C. The samples were run in duplicate. For each sample, mRNA expression levels for specific transcripts were normalized to the amount of glyceraldehyde-3-phosphate dehydrogenase (GAPDH). The primer sequences are shown in Table 1.

**Table 1:** Primers used for real-time PCR.

Gene	Forward	Reverse
TLR4	GCTTTCACCTCTGCCTTCAC	AGGCGATACAATTCCACCTG
TNF-α	GTGGAACTGGCAGAAGAG	CCATAGAACTGATGAGAGG
IL-1β	CTGTGTCTTTCCCGTGGACC	CAGCTCATATGGGTCCGACA
IL-6	TTCCATCCAGTTGCCTTCTTGG	TTCTGCAAGTGCATCATCG
iNOS	TGACGCTCGGAACTGTAGCAC	TGATGGCCGACCTGATGTT
COX-2	GATGACTGCCCAACTCCC	AACCCAGGTCCTCGCTTA
GAPDH	TGCACCACCAACTGCTTAGC	GGATGCAGGGATGATGTTCTG

### 2.3 Enzyme-linked immunosorbent assay (ELISA)

The protein levels of TNF-α, IL-1β, and IL-6 in the culture media were measured by mouse TNF-α, IL-1β, and IL-6 Quantikine® ELISA kit (R&D Systems, Minneapolis, MN, USA). Briefly, the assay diluent was added into 96-well plate followed by diluted standards, control and samples for 2 h incubation at room temperature. After washed five times with wash buffer, mouse TNF-α, IL-1β or IL-6 conjugate were added and incubated for another 2 h. The substrate solution was then added to the plate after washed with wash buffer. The reaction was stopped using stop solution after 30 min. The optical density was detected using a microplate reader (Tecan Infinite 200, Männedorf, Switzerland) at 450 nm with wavelength correction at 540 nm. The results were calculated based on the standard curve.

### 2.4 Cell viability

A cell viability assay was conducted using the Cell-Counting Kit-8 (CCK-8; Dojindo Molecular Technologies, Inc., Kumamoto, Japan). MG6 cells were pre-incubated with or without AIII (100 μM) for 3 h, followed by the addition of LPS (5 ng/mL) and/or AIII (100 μM) for another 3 h. After 1 h incubation with the reagent, the optical density was measured at 450 nm with a reference length of 540 nm using a microplate reader (Tecan Infinite 200).

### 2.5 Western blotting analysis

Protein levels of TLR4 and the phosphorylation of p38 MAPK, JNK, and NF-κB in MG6 cells were analyzed by Western blotting, as previously described [16]. The whole cells were lysed by RIPA buffer (150 mM NaCl, 50 mM Tris, 5 mM EDTA, 50 mM NaF, 10 mM sodium pyrophosphate, 1 mM sodium orthovanadate, 1% NP-40, 0.5% deoxycorate, pH 8.0 supplemented with 1 mM leupeptin, 1 mM phenylmethylsulfonyl fluoride and 1 μg/mL aprotinin), scraped off and collected for analysis. Lysates were homogenized, sonicated for 20 s, added with 1% Triton-X and 0.2% SDS followed by incubation for 30 min at rotary shaker (24 °C, 100 rpm). Supernatants were collected after centrifuged at 15,000 × g for 15 min at 4 °C. Forty-five microliter sample lysates were boiled for 5 min with 50 μl of Tris-Glycine SDS sample buffer 2X and 5 μl 2-mercaptoethanol.

Samples containing 20 μg proteins were loaded on 8–16% Tris-Glycine Gel (Invitrogen, Massachusets, USA) and transferred onto PVDF membrane (Novex, California, USA). The membrane was blocked with Tris-buffered saline and 0.1% Tween 20 (TBST) supplemented with 5% skim milk powder for 10 min, followed by overnight incubation at 4 °C with primary antibodies. The membrane then washed three times with TBST, and incubated with secondary antibodies for 1 h at room temperature. Protein levels of TLR4 were normalized to GAPDH. The primary antibodies used in this study are shown in Table 2. The secondary antibodies were used an anti-rabbit and anti-mouse IgG antibody conjugated to horseradish peroxidase (1:1000; GE HealthCare UK Ltd., Buckinghamshire, UK). Blots were detected using Amersham ECL (GE HealthCare UK Ltd.), analyzed with LAS-1000 image analyzer (Fujifilm, Tokyo, Japan), and quantified by Image J (National Institute of Health, MD, USA).

**Table 2:** Primary antibodies used for Western blotting analysis.

Antigen	Type	Dilution	Catalog No.	Manufacturer
TLR4	MM	1:100	sc-293072	Santa Cruz Biotechnology, CA, USA
GAPDH	MM	1:2000	sc-32233	Santa Cruz Biotechnology, CA, USA
p38 MAPK	RP	1:1000	9212S	Cell Signaling Technology, MA, USA
Phospho-p38 MAPK	RP	1:1000	9211S	Cell Signaling Technology, MA, USA
JNK	RP	1:1000	9252S	Cell Signaling Technology, MA, USA
Phospho-JNK	RP	1:250	9251S	Cell Signaling Technology, MA, USA
NF-κB	RM	1:50000	8245S	Cell Signaling Technology, MA, USA
Phospho-NF-κB	RM	1:50000	3033S	Cell Signaling Technology, MA, USA

MM: mouse monoclonal antibody; RP: rabbit polyclonal antibody; RM: rabbit monoclonal antibody.

### 2.6 Statistical analysis

Results are expressed as mean ± standard error of the mean (SEM) and analyzed with GraphPad Prism version 4.0 software (GraphPad Software, La Jolla, CA, USA) using one-way ANOVA followed by the Tukey-Kramer test with *p* < 0.05 considered significant.

## 3 Results

### 3.1 Effects of AIII on TLR4 expression in MG6 cells

**Figure 1:**
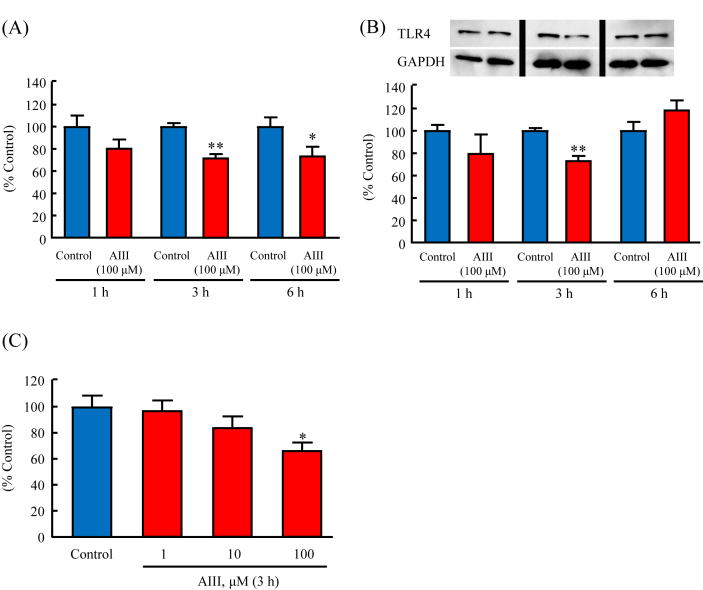
Effects of AIII on TLR4 expression in MG6 cells. Cells were cultured for 1, 3, and 6 h in the absence or presence of AIII (100 μΜ). The mRNA expression of TNF-α (A) was examined by RT-PCR and the protein levels of TLR4 (B) were examined by Western blotting analysis. Non-adjusted images of Western blotting analysis were presented in Supplementary Material Figure 1. (C) Concentration dependence of AIII on TLR4 mRNA expression was examined by RT-PCR after the cells were incubated with AIII (1, 10, and 100 μM) for 3 h. Results are expressed as the mean percentage ±SEM. ∗p < 0.05, ∗∗p < 0.01 compared with the control group, n = 9–15.

The time course analysis of TLR4 mRNA expression showed that AIII (100 μM) significantly reduced TLR4 mRNA expression at 3 h and 6 h to 72% and 73%, respectively, of that in the control group (Figure 1A). Western blotting analysis also revealed that treatment of MG6 cells with AIII (100 μM) for 3 h significantly reduced TLR4 protein levels to 73% of that in the control group (Figure 1B). Furthermore, MG6 cells treated with AIII at different concentrations and incubated for 3 h exhibited attenuated TLR4 mRNA expression, with 100 μM of AIII significantly reducing TLR4 mRNA expression to 66% of that in the control group (Figure 1C). Therefore, we treated the cells with AIII at 100 μM for 3 h in the following experiments.

### 3.2 Effects of AIII on mRNA expression and protein levels of TNF-α, IL-1β, IL-6, iNOS, and COX-2 induced by LPS in MG6 cells and RAW264.7 cells

**Figure 2:**
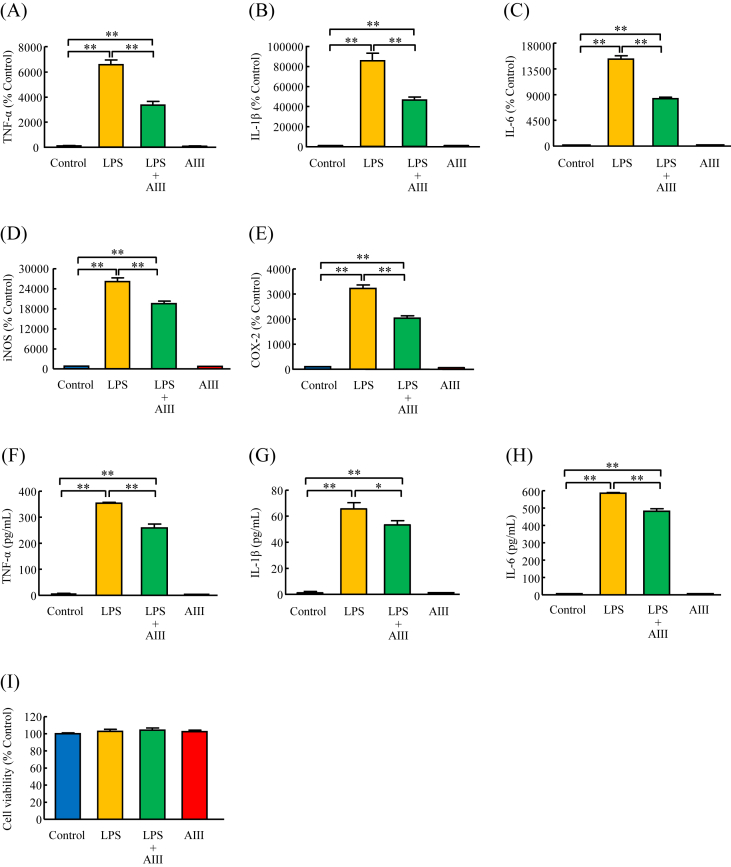
Effects of AIII on mRNA expression and protein levels of TNF-α, IL-1β, IL-6, iNOS, and COX-2 induced by LPS in MG6 cells. Cells were cultured for 3 h in the absence or presence of AIII (100 μΜ), followed by the addition of LPS (5 ng/mL) for another 3 h. The mRNA expression of TNF-α (A), IL-1β (B), IL-6 (C), iNOS (D), and COX-2 (E) was examined by RT-PCR. The culture media were collected for analysis of the protein levels of TNF-α (F), IL-1β (G), and IL-6 (H) by ELISA. (I) Effects of AIII on MG6 cell viability was examined by CCK-8 assay. Results are expressed as mean ± SEM. ∗p < 0.05, ∗∗p < 0.01, n = 6–9.

In MG6 cell cultures, pretreatment with AIII (100 μM) for 3 h significantly decreased the LPS (5 ng/mL)-induced increase in the mRNA expression of TNF-α, IL-1β, and IL-6 to 51%, 54%, and 55%, respectively, of that in the LPS-treated group (Figure 2A, B, C). Pretreatment of MG6 cells with AIII (100 μM) also significantly suppressed mRNA expression of iNOS and COX-2 induced by LPS (5 ng/mL) to 75% and 63%, respectively, of that in the LPS-treated group (Figure 2D and E). Next, the concentrations of TNF-α, IL-1β and IL-6 released in the culture media were measured by ELISA. Consistent with the mRNA expression results, pretreatment of MG6 cells with AIII (100 μM) for 3 h significantly suppressed the protein levels of TNF-α, IL-1β and IL-6 induced by LPS (5 ng/mL) to 73%, 81%, and 82%, respectively, of that in the LPS-treated group (Figure 2F, G, H). In addition, the CCK-8 assay was performed to examine the potential cytotoxic effects of AIII on MG6 cells. As shown in Figure 2I, treatment of MG6 cells with either AIII (100 μM) or with LPS (5 ng/mL) did not significantly affect cell viability compared with the control group.

**Figure 3:**
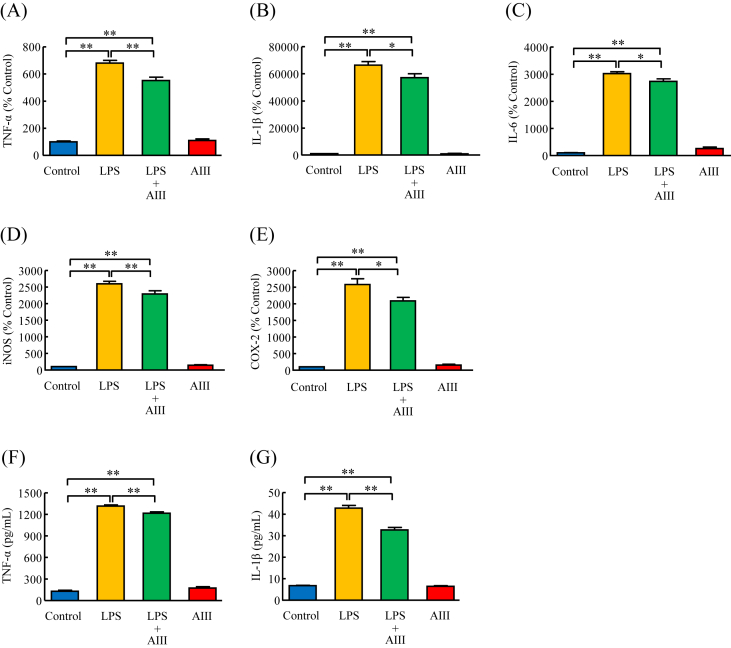
Effects of AIII on mRNA expression and protein levels of TNF-α, IL-1β, IL-6, iNOS, and COX-2 induced by LPS in RAW264.7 cells. Cells were cultured for 3 h in the absence or presence of AIII (100 μΜ), followed by the addition of LPS (5 ng/mL) for another 3 h. The mRNA expression of TNF-α (A), IL-1β (B), IL-6 (C), iNOS (D), and COX-2 (E) was examined by RT-PCR. The culture media were collected for analysis of the protein levels of TNF-α (F) and IL-1β (G) by ELISA. Results are expressed as mean ± SEM. ∗p < 0.05, ∗∗p < 0.01, n = 9.

Pretreatment of RAW264.7 cells with AIII (100 μM) for 3 h significantly decreased the LPS (5 ng/mL)-induced increase in the mRNA expression of TNF-α, IL-1β, IL-6, iNOS, and COX-2 to 81%, 86%, 90%, 88% and 81% respectively, of that in the LPS-treated group (Figure 3A, B, C, D, E). Furthermore, pretreatment of RAW264.7 cells with AIII (100 μM) for 3 h significantly inhibited the increase in the protein levels of TNF-α and IL-1β induced by LPS (5 ng/mL) to 92% and 76%, respectively, of that in the LPS-treated group (Figure 3F and G).

### 3.3 Effects of AIII on mRNA expression and protein levels of TNF-α, IL-1β, IL-6, iNOS, and COX-2 induced by LPS in PMC

**Figure 4:**
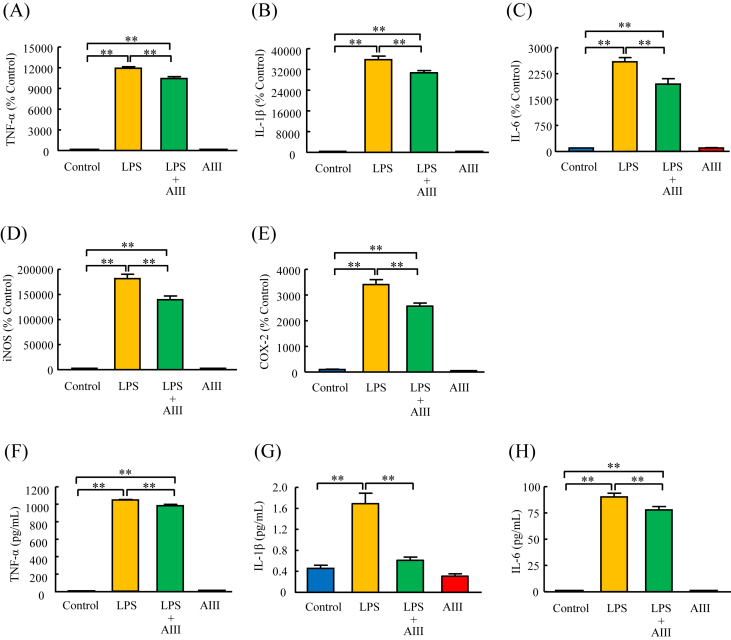
Effects of AIII on mRNA expression and protein levels of TNF-α, IL-1β, IL-6, iNOS, and COX-2 induced by LPS in PMC. Cells were cultured for 3 h in the absence or presence of AIII (100 μΜ), followed by the addition of LPS (5 ng/mL) for another 3 h. The mRNA expression of TNF-α (A), IL-1β (B), IL-6 (C), iNOS (D), and COX-2 (E) was examined by RT-PCR. The culture media were collected for analysis of the protein levels of TNF-α (F), IL-1β (G), and IL-6 (H) by ELISA. Results are expressed as mean ± SEM. ∗∗p < 0.01, n = 6–8.

We further examined the effects of AIII on cytokine and enzyme production in PMC. Similar to the results obtained in MG6 cells (Figure 2), pretreatment of PMC with AIII (100 μM) for 3 h significantly reduced the LPS (5 ng/mL)-induced increase in mRNA expression of TNF-α, IL-1β, and IL-6 to 88%, 86%, and 75%, respectively, of that in the LPS-treated group (Figure 4A, B, C). Pretreatment of PMC with AIII (100 μM) also significantly reduced the mRNA expression of iNOS and COX-2 induced by LPS (5 ng/mL) to 77% and 75%, respectively, of that in the LPS-treated group (Figure 4D and E). Moreover, pretreatment of PMC with AIII (100 μM) for 3 h significantly decreased the protein levels of TNF-α, IL-1β, and IL-6 induced by LPS (5 ng/mL) to 94%, 36%, and 86%, respectively, of that in the LPS-treated group (Figure 4F, G, H).

### 3.4 Effects of AIII on the phosphorylation of p38 MAPK, JNK, and NF-κB induced by LPS in MG6 cells

**Figure 5:**
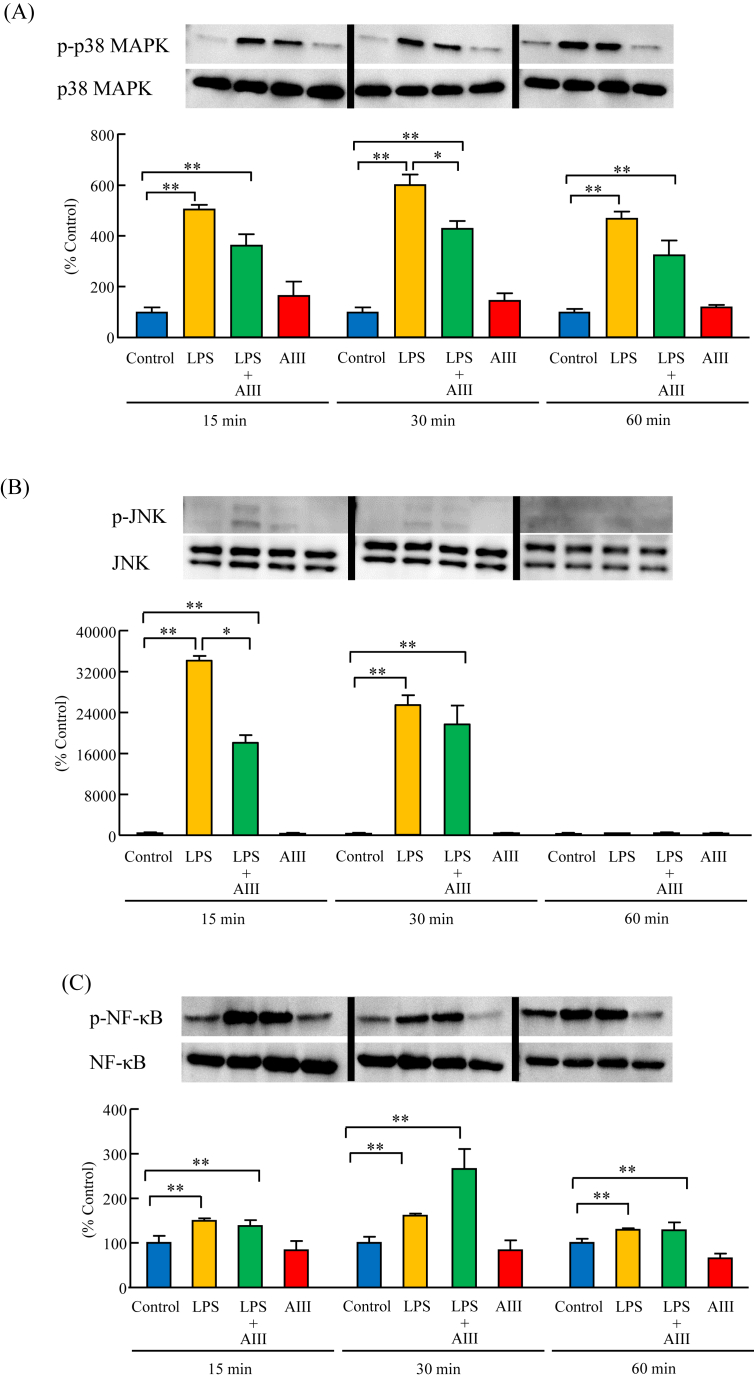
Effects of AIII on the phosphorylation of p38 MAPK, JNK, and NF-κB induced by LPS in MG6 cells. Cells were cultured for 3 h in the absence or presence of AIII (100 μΜ), followed by the addition of LPS (5 ng/mL) for 15, 30, and 60 min. The phosphorylation of p38 MAPK (A), JNK (B), and NF-κB (C) was examined by Western blotting analysis. Non-adjusted images of Western blotting analysis were presented in Supplementary Material Figure 2. Results are expressed as mean ± SEM. ∗p < 0.05, ∗∗p < 0.01, n = 3.

The phosphorylation of p38 MAPK, JNK, and NF-κB induced by LPS (5 ng/mL) after pretreatment of MG6 cells with AIII (100 μM) was examined by Western blotting analysis. Pretreatment of MG6 cells with AIII for 3 h significantly inhibited the LPS (5 ng/mL)-induced phosphorylation of p38 MAPK at 30 min (Figure 5A) and the phosphorylation of JNK at 15 min (Figure 5B). Pretreatment of MG6 cells with AIII, however, did not affect the phosphorylation of NF-κB induced by LPS (5 ng/mL; Figure 5C).

## 4 Discussion

In the present study, we demonstrated the anti-inflammatory effects of AIII on LPS-induced inflammation in mouse microglia. Our results showed that AIII significantly inhibited the mRNA expression and protein levels of TLR4 in MG6 cells. Furthermore, AIII pretreatment significantly inhibited the mRNA expression and protein levels of TNF-α, IL-1β, and IL-6, and inflammatory enzymes including iNOS and COX-2 induced by LPS in MG6 cells and PMC with no cytotoxic effects. Moreover, AIII pretreatment of MG6 cells significantly suppressed the activation of p38 MAPK and JNK following LPS stimulation. These findings demonstrated that AIII downregulated TLR4 expression, inhibited the p38 MAPK and JNK pathways, and subsequently suppressed the LPS-induced production of cytokines and enzymes in mouse microglia.

Previously, it was reported that in vitro treatment of mouse peritoneal macrophages with AIII significantly decreased LPS-induced TNF-α and nitric oxide (NO) production [2]. AIII also suppressed the release of pro-inflammatory cytokines, and COX-2 through the inactivation of NF-κB and MAPK pathways following LPS stimulation in RAW264.7 mouse macrophages [3]. Consistent with these earlier results, our findings demonstrated that AIII significantly inhibited the increases in the protein levels and mRNA expression of TNF-α, IL-1β, IL-6, iNOS, and COX-2 induced by LPS in RAW264.7 cells. Taken together, these findings indicate that AIII exhibits anti-inflammatory effects in the peripheral system. The effects of AIII and underlying mechanisms of the effects on brain inflammation, however, remained to be elucidated. In this regard, we examined how AIII influences LPS responses in mouse microglia. It is well established that LPS strongly activates microglia through TLR4, leading to the production of pro-inflammatory cytokines such as TNF-α, IL-1β, and IL-6, and inflammatory enzymes, such as iNOS and COX-2 [9, 10, 11]. The overproduction and exaggerated release of these molecules is linked to the development of neuropathic pain, psychiatric disorders, and neurodegenerative diseases [9, 17, 18]. TNF-α, IL-1β, and IL-6 are involved in the progression of brain inflammation through inducing the expression of chemokines that facilitate the infiltration of leukocytes into the CNS [17]. These cytokines are also required for the activation of NO and prostaglandin E2 (PGE2) production in LPS-stimulated microglia [19]. High levels of NO and PGE2 produced by iNOS and COX-2 have been defined as cytotoxic molecules and contribute to the pathogenesis of endotoxin shock [20, 21]. iNOS is not normally expressed in the brain, but it is expressed in the inflammatory state following microglia stimulation by LPS [19]. COX-2 is an inducible enzyme whose expression is increased in areas of inflammation as a response to inflammatory stimuli, including LPS insult. Previous studies indicated that COX-2 overproduction correlates well with cytotoxicity in the brain and its inhibition reduces brain injury after ischemia and slows the progression of AD and PD [22]. Therefore, excess production of these pro-inflammatory cytokines and enzymes is a hallmark of brain inflammation, and inhibiting their production may facilitate the control of neuroinflammatory disorders. In this study, we demonstrated that AIII exerts downregulation effects on TLR4 expression in MG6 cells and significantly suppressed the LPS-induced mRNA expression and protein release of TNF-α, IL-1β, IL-6, iNOS, and COX-2 in MG6 cells and PMC without cytotoxicity. The mechanism by which AIII regulates TLR4 expression, however, remains unclear. Reduction of TLR4 expression by AIII renders microglia less sensitive to LPS-induced activation, thus inhibiting the LPS-induced production of pro-inflammatory cytokines and enzymes in microglia. Because the findings in the present study were obtained in in vitro experiments, the in vivo study should be conducted to elucidate the precise action of AIII on brain inflammation.

LPS is reported to activate the MAPKs (p38, JNK, and ERK1/2) and the NF-κB pathway via TLR4 in microglia. These pathways are involved in the production of pro-inflammatory cytokines, including TNF-α, IL-1β, and IL-6, as well as iNOS and COX-2 [9, 10, 11,21]. Therefore, we further examined whether the inhibitory effects of AIII on the LPS-induced production of cytokines and enzymes in MG6 cells were due to the inactivation of MAPKs and NF-κB pathways. In this study, pretreatment of MG6 cells with AIII significantly attenuated the LPS-induced phosphorylation of MAPK pathways, in particular the p38 MAPK and JNK, without affecting the phosphorylation of NF-κB. These results clearly showed that the anti-inflammatory activities of AIII are strongly linked to the suppression of p38 MAPK and JNK in the LPS-stimulated microglia. In agreement with our results, Uesugi et al. [23] revealed that p38 MAPK and JNK, but not NF-κB, are crucial for the LPS-induced production of TNF-α in microglia. In particular, a previous study demonstrated that p38 MAPK and JNK are important pathways contributing to glia-induced neuronal death and correlate well with neurodegenerative diseases, including AD, PD, and brain ischemia [24]. Other studies further demonstrated that blockade of the p38 MAPK and JNK pathways with a specific inhibitor in microglia led to the inhibition of pro-inflammatory cytokines, chemokines, and enzymes [25, 26]. In contrast to our findings, Ji et al. [3] reported that AIII has anti-inflammatory effects through suppressing the release of pro-inflammatory cytokines related to the NF-κB and MAPK pathways. The differences in the experimental schedule, dosage of LPS, and type of cell being used might be responsible for this discrepancy. On the other hand, in the present study, the addition of LPS (5 ng/mL) did not affect the phosphorylation of ERK 1/2 in MG6 cells (data not shown). This result may be due to the low concentration of LPS used. Horvath et al. [27] demonstrated no discernible increase in the phosphorylation of ERK 1/2 in immortalized microglia cell lines, BV-2 cells and HAPI cells, following stimulation with 1 μg/mL - 5 μg/mL LPS. Consistent with this finding, a study using MG6 cells demonstrated that stimulation with 50–100 ng/mL LPS, but not treatment with 10 ng/mL LPS, induced ERK 1/2 phosphorylation [28]. These findings provide compelling evidence that AIII exerts anti-inflammatory effects through inhibition of p38 MAPK and JNK pathways, leading to the suppression of LPS-induced TNF-α, IL-1β, IL-6, iNOS, and COX-2 production in microglia.

In summary, our results indicate that AIII significantly downregulates TLR4 expression. This downregulation, at least in part, leads to inactivation of the p38 MAPK and JNK pathways and ultimately suppresses LPS-induced increases in TNF-α, IL-1β, IL-6, iNOS, and COX-2 mRNA expression and protein levels in microglia. However, further studies in other glial cells in the CNS and even in vivo researches are necessary to be attempted, which can intensively determine the impact of AIII on brain inflammation models.

## Declarations

### Author contribution statement

Goro Katsuura: Performed the experiments; Wrote the paper.

Namiko Kawamura: Performed the experiments; Analyzed and interpreted the data.

Akihiro Asakawa, Akio Inui: Contributed reagents, materials, analysis tools or data.

### Funding statement

A. Inui was supported by a Grant-in-Aid for General Scientific Research from the Ministry of Education, Culture, Sports, Science, and Technology (MEXT) in Japan (No. 16H06404).

E. Novianti was supported by a scholarship of the Program for Research and Innovation in Science and Technology (RISET-Pro), provided by the 10.13039/501100010447Ministry of Research, Technology and Higher Education of the Republic of Indonesia.

G. Katsuura was supported by a grant from the 10.13039/501100004330Smoking Research Foundation Japan.

### Data availability statement

Data will be made available on request.

### Declaration of interests statement

The authors declare no conflict of interest.

### Additional information

No additional information is available for this paper.
