# Supplementary material for: Atractylenolide-III suppresses lipopolysaccharide-induced inflammation via downregulation of toll-like receptor 4 in mouse microglia
Source: Heliyon. 2021 Oct 27;7(10):e08269. doi: 10.1016/j.heliyon.2021.e08269 (PMC8569437; doi:10.1016/j.heliyon.2021.e08269)

Supplementary Material Figure 1.

Non-adjusted images of the Western blotting analysis in Figure 1B.

(A)

TLR4

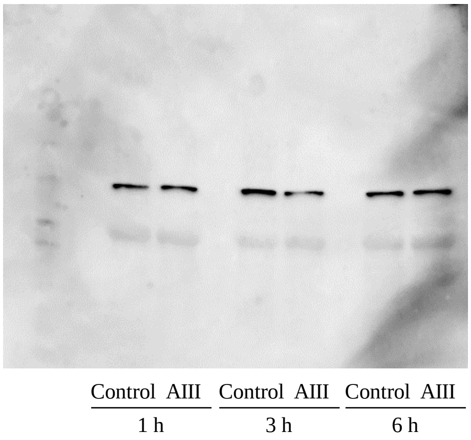

(B)

GAPDH

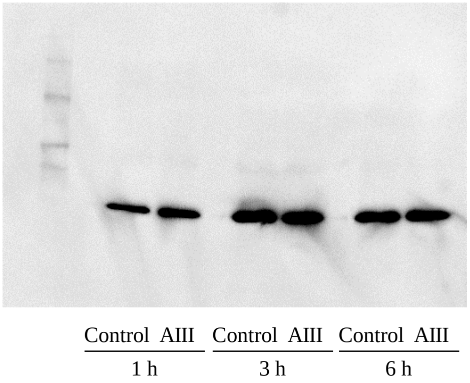

Supplementary Material Figure 2.

Non-adjusted images of the Western blotting analysis in Figure 5.

(A)

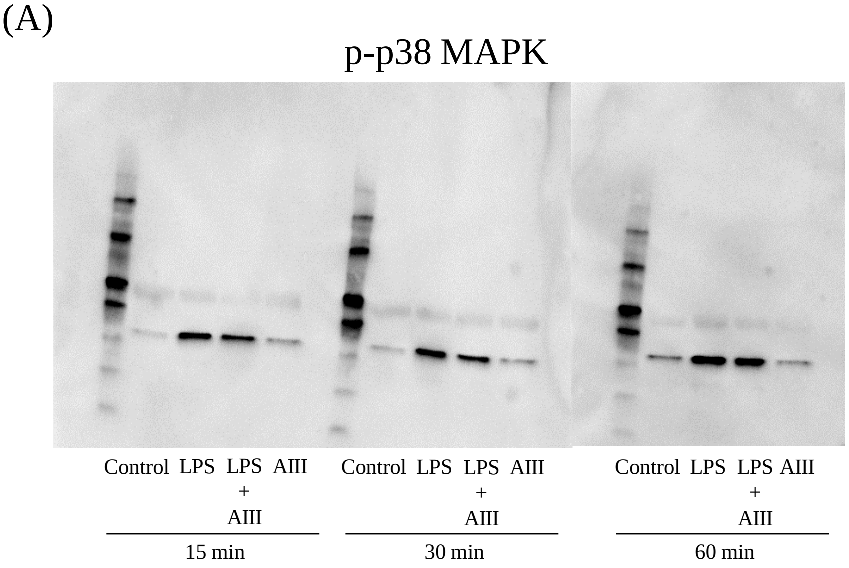

(B)

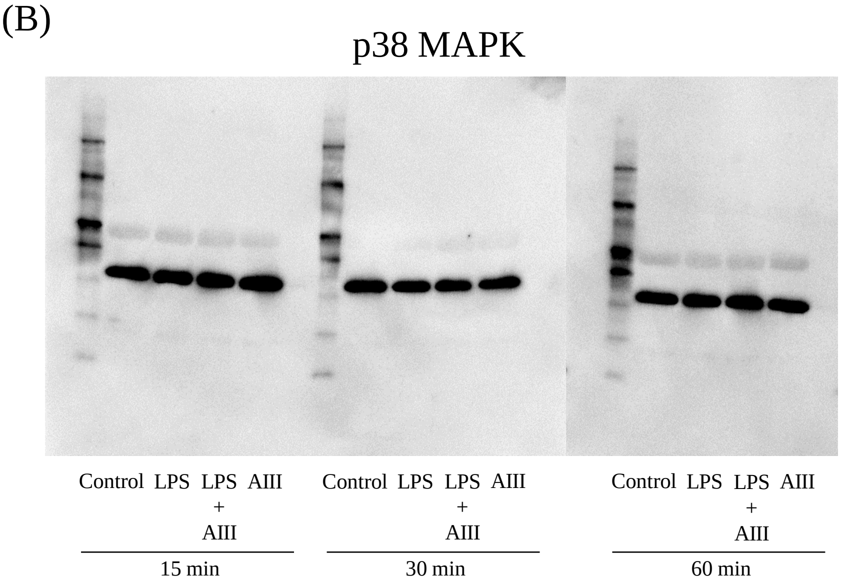

Supplementary Material Figure 2.

Non-adjusted images of the Western blotting analysis in Figure 5.

(C)

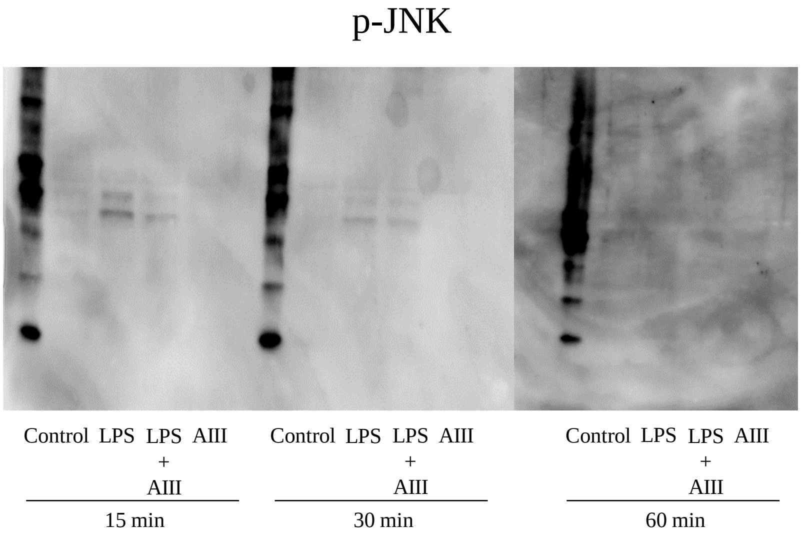

(D)

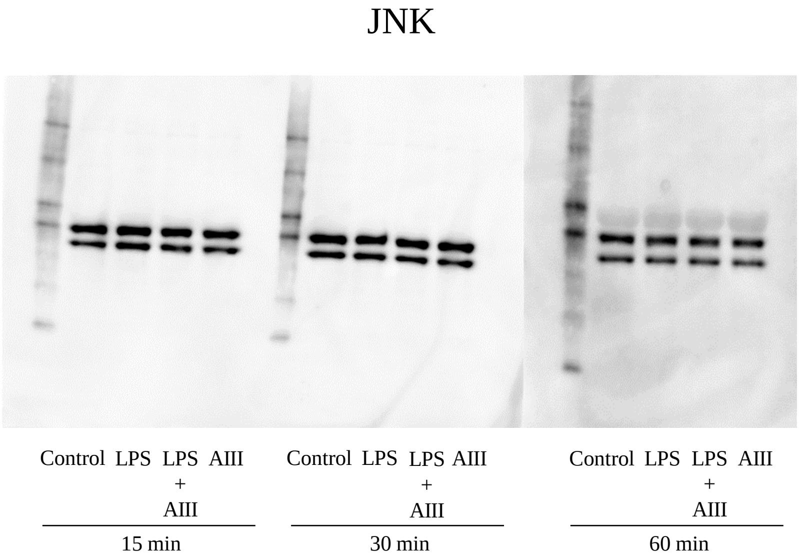

Supplementary Material Figure 2.

Non-adjusted images of the Western blotting analysis in Figure 5.

(E)

p-NF-κB

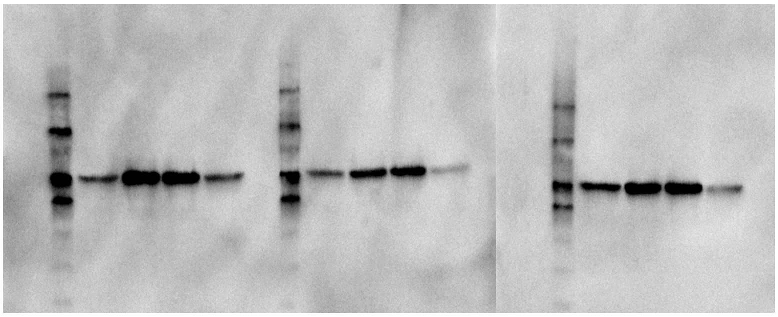

(F)

NF-κB

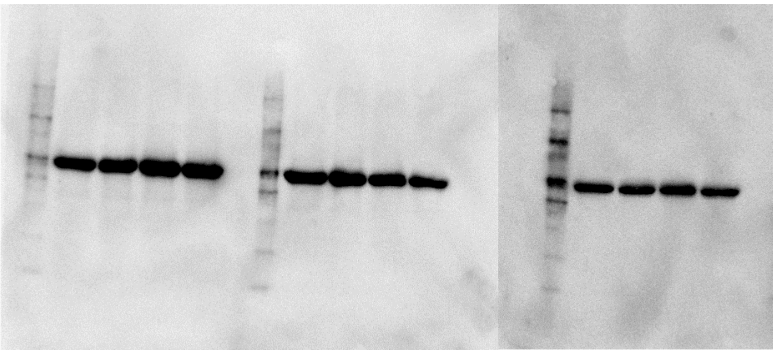

Supplement: Supplementary file 1 — Supplementary Material Figure [file mmc1.pdf]
